# Ancient dental pulp: Masterpiece tissue for paleomicrobiology

**DOI:** 10.1002/mgg3.1202

**Published:** 2020-03-31

**Authors:** Ba Hoang Anh Mai, Michel Drancourt, Gérard Aboudharam

**Affiliations:** ^1^ Aix‐Marseille Université IRD MEPHI IHU‐Méditerranée Infection Marseille France; ^2^ Hue University of Medicine and Pharmacy Thua Thien Hue Vietnam; ^3^ UFR Odontologie Aix‐Marseille Université Marseille France

**Keywords:** ancient dental pulp, infectious diseases, nucleic acids, paleomicrobiology, proteins

## Abstract

**Introduction:**

Dental pulp with special structure has become a good reference sample in paleomicrobiology‐related blood‐borne diseases, many pathogens were detected by different methods based on the diagnosis of nucleic acids and proteins.

**Objectives:**

This review aims to propose the preparation process from ancient teeth collection to organic molecule extraction of dental pulp and summary, analyze the methods that have been applied to detect septicemic pathogens through ancient dental pulps during the past 20 years following the first detection of an ancient microbe.

**Methods:**

The papers used in this review with two main objectives were obtained from PubMed and Google scholar with combining keywords: “ancient,” “dental pulp,” “teeth,” “anatomy,” “structure,” “collection,” “preservation,” “selection,” “photography,” “radiography,” “contamination,” “decontamination,” “DNA,” “protein,” “extraction,” “bone,” “paleomicrobiology,” “bacteria,” “virus,” “pathogen,” “molecular biology,” “proteomics,” “PCR,” “MALDI‐TOF,” “LC/MS,” “ELISA,” “immunology,” “immunochromatography,” “genome,” “microbiome,” “metagenomics.”

**Results:**

The analysis of ancient dental pulp should have a careful preparation process with many different steps to give highly accurate results, each step complies with the rules in archaeology and paleomicrobiology. After the collection of organic molecules from dental pulp, they were investigated for pathogen identification based on the analysis of DNA and protein. Actually, DNA approach takes a principal role in diagnosis while the protein approach is more and more used. A total of seven techniques was used and ten bacteria (*Yersinia pestis*, *Bartonella quintana*, *Salmonella enterica serovar Typhi*, *Salmonella enterica serovar Paratyphi C*, *Mycobacterium leprae*, *Mycobacterium tuberculosis*, *Rickettsia prowazeki*, *Staphylococcus aureus*, *Borrelia recurrentis*, *Bartonella henselae*) and one virus (*Anelloviridae)* were identified. *Y. pestis* had the most published in quantity and all methods were investigated for this pathogen, *S. aureus* and *B. recurrentis* were identified by three different methods and others only by one. The combining methods interestingly increase the positive rate with ELISA, PCR and iPCR in *Yersinia pestis* diagnosis. Twenty‐seven ancient genomes of *Y. pestis* and one ancient genome of *B. recurrentis* were reconstructed. Comparing to the ancient bone, ancient teeth showed more advantage in septicemic diagnosis. Beside pathogen identification, ancient pulp help to distinguish species.

**Conclusions:**

Dental pulp with specific tissue is a suitable sample for detection of the blood infection in the past through DNA and protein identification with the correct preparation process, furthermore, it helps to more understand the pathogens of historic diseases and epidemics.

## Graphical abstract

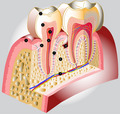

## 1 INTRODUCTION

Based primarily on the amplification and sequencing of ancient DNA (aDNA), the development of techniques for the detection, identification, and characterization of ancient pathogens has allowed us to better understand the natural history of some infectious diseases and their evolution as well as provide increasing knowledge on ancient microbiota and resolve historical controversies (Drancourt et al., 2007). In 1998, for the first time, *Yersinia pestis* was detected by molecular biology in human dental pulps collected from skeletons buried in French graves dating back to the 18th century. This research opened a new way to establish the etiology of ancient infectious diseases from human or animal remains (Drancourt, Aboudharam, Signoli, Dutour, & Raoult, 1998). Furthermore, two experimental studies confirmed the interest of using dental pulp as a potential reservoir of blood‐borne bacteria. These works were conducted on guinea pigs by causing a bacteremia with *Coxiella burnetii*. The first work showed that *C. burnetii* DNA was detected after a long time in the dental pulp, whereas it was no longer present in blood and spleen. This late detection was called the "memory effect" of the dental pulp (Aboudharam, Lascola, Raoult, & Drancourt, 2000). The second work indicated the persistence of live bacteria in the dental pulp after intraperitoneal infection (Aboudharam, Drancourt, & Raoult, 2004). The dental pulp is a conjunctive soft tissue, derived from ectomesenchyme, containing an abundant vascular system and nerves, in which anastomoses between venules and arterioles allow regular blood flow pressure. Therefore, microorganisms could penetrate and circulate in blood vessels and colonize the dental pulp (Dang, Aboudharam, Drancourt, & Raoult, 2008). In contrast to other tissues collected from ancient corpses and skeletons, such as hair, bones, mummified tissues that were exposed to the environment and more easily degraded; the dental pulp is located into a close cavity, isolated and well‐protected from the outside by enamel and dentin. Enamel and dentin are very hard mineralized tissues. They resist to external degradation factors and are the parts that last the longest after degradation of the body (Higgins & Austin, 2013; Krishan, Kanchan, & Garg, 2015). The dental pulp has become ultimately a reference tool for research in paleomicrobiology and the establishment of new knowledge for the history of some infectious diseases. Many international research teams now use ancient dental pulp with different methods based essentially on DNA (amplification/sequencing) and protein analysis to detect ancient septicemic agents.

## 2 METHODS

The papers used in this review were obtained from PubMed and Google scholar. To establish a standard procedure for using ancient teeth in paleomicrobiology, we used keywords or in combination: “ancient,” “teeth,” “dental pulp,” “anatomy,” “structure,” “preservation,” “selection,” “photography,” “radiography,” “collection,” “contamination,” “decontamination,” “DNA,” “protein,” “extraction”. To identify pathogen and methods, we used terms or in combination including “ancient,” “teeth,” “dental pulp,” “bone,” “paleomicrobiology,” “bacteria,” “virus,” “pathogen,” “DNA,” “molecular biology,” “protein,” “proteomics,” “PCR,” “MALDI‐TOF,” “LC/MS,” “ELISA,” “immunology,” “genome,” “microbiome,””immunochromatography,” “metagenomics.”

## 3 RESULTS

### 3.1 Preparation process

#### 3.1.1 Collection

After death, the human body decomposes, this process can be modified depending on the burial conditions, natural postmortem degradation, and environmental conditions (Wills, Ward, & Vanessa, 2014). When human remains are found in archaeological sites, the anthropologist carefully collects samples to avoid any contamination, principles such as using facemask, head‐dress gown, lab coat, disposable gloves, and sterile instruments should be implemented (Rizzi, Lari, Gigli, De Bellis, & Caramelli, 2012). It is important to keep the teeth in their alveolus on the jawbone for better protection and to decrease the risk of contamination (Dang et al., 2008; De Leo, Turrina, & Marigo, 2000). Samples are usually preserved in archeology laboratories. The teeth with or without jawbone can be addressed to other laboratories for various studies.

#### 3.1.2 Preservation

It has been established that the environmental conditions, especially the temperature, greatly influence the degradation of DNA in ancient teeth (Alvarez García et al., 1996). Following an experimental study of teeth burial, we noticed that an increase of 2°C in soil could divide by two the half‐life of mtDNA and nuclear fragments. This evidence demonstrated the significant impact of temperature on the yield of DNA in dental tissues. Thus, storage of teeth samples at a low temperature would minimize the rate of DNA degradation and help make genetic analysis more accurate (Higgins, Rohrlach, Kaidonis, Townsend, & Austin, 2015). Preferable conditions of preservation for ancient samples would be a dry environment and a temperature of −20° (Rizzi et al., 2012).

#### 3.1.3 Selection

Human teeth are divided into four groups with different sizes and shapes, yet the histological structure of all teeth is almost identical (Malaver & Yunis, 2003). The volume of the pulp is correlated with the amount of DNA recovered, the larger the pulp volume, the more the pulp cavity contains cells (De Leo et al., 2000). Teeth selection for pulp investigation should preferably focus on larger pulp cavities. Indeed, our unpublished data on the volume of old teeth indicates a significant difference between the total volume of the teeth and the volume of the pulp of the four types of teeth. The latter is, from the largest to the smallest, molars, canines, premolars, and incisors (Table 1). A guideline for suitable ancient teeth selection was proposed in order to obtain as much dental pulp as possible, it describes several possibilities (Dang et al., 2008): (a) intact teeth with a closed apex, (b) teeth with single root are easier to manipulate and may have a larger cavity and yield a larger volume of pulp, (c) unerupted teeth should be investigated instantly after being removed from the jawbone, (d) multiple teeth were chosen per individual to maximize chances of detection. For example, the number of ancient teeth per individual analyzed by PCR and containing the plague agent *Y. pestis* was 3/3 teeth, 1/4 teeth, and 1/2 teeth (Drancourt et al., 1998). Also, after two teeth were extracted from the same individual, PCR analysis revealed that the first contained both *B. quintana* and *Y. pestis*, the second contained only *Y. pestis* (Tran, Forestier, Drancourt, Raoult, & Aboudharam, 2011).

**TABLE 1:** Pulp volume of each teeth type from nine ancient male mandibles was measured by CT‐Scanner (unpublished data)

Teeth type	*n*	Pulp volume Mm^3^	*p*
Incisors	32	12.4 ± 3.3	<.05
Canines	17	36.1 ± 10.2	
Premolars	35	24.1 ± 7.2	
Molars	35	56.7 ± 13.2	

The age of teeth before extraction from the jawbone or death has a significant impact on nuclear DNA yield, as the older teeth resist better to decomposition. This observation can be explained by the dental structure. Indeed, mature teeth have closed apices, more compact enamel, and dentin, thicker and less porous cementum, therefore DNA in these teeth is preserved and less degraded than in young teeth (Higgins et al., 2015)*.*

#### 3.1.4 Digital photography

This stage aims at recording the visual information of teeth such as their form, color, fissures, and position on the jaws (Dang et al., 2008). Teeth and jaws should be photographed with different orientations to obtain a complete overview. Currently, we photograph the four sides of the teeth when they are dissociated from the jaw. However, the use of a scale would allow comparing the size on the photos or allow re‐specifying the measure when necessary.

#### 3.1.5 Radiography

Dental X‐ray is used to visualize the internal structure of the teeth, such as the pulp chamber, the presence of calcification, sometimes detect discrete fissures on teeth and jaws. Advanced imaging technologies including Magnetic Resonance Imaging (MRI), Computed Tomography (CT) scan, spiral CT, cone‐beam CT, micro‐CT and Synchrotron Radiation‐based micro‐Computed Tomography (SRµCT) have been widely employed to yield the detailed image of the complex dental structure. Furthermore, these techniques allowed for an easier and more accurate analysis (Kato, Ziegler, Utsumi, Ohno, & Takeichi, 2016). The accumulation of X‐ray irradiation influences the degradation of aDNA, indeed, the quality of aDNA from bone samples is unaffected with doses below 200 Gy, negligible between 200 Gy and 2.000 Gy, but the quality of the DNA is deteriorating with doses above 2.000 Gy (Immel et al., 2016).

#### 3.1.6 Decontamination

The contamination with exogenous sources from the archaeological context or laboratory manipulation is a major issue when working with ancient skeletons***,*** reducing contamination risks is an important challenge for the aDNA study to ensure authentic results. Environmental DNA could penetrate into the samples, depending on their humidity (Sampietro et al., 2006), for ancient teeth, contaminants can be found at the level of the apex. After washing extensively with sterile water and removing any retained soft tissue or bone adherence, teeth could be decontaminated with different techniques such as the removal of the outer layer by a high‐speed surgical handpiece (Rubio, Martinez, Martinez, & Martin de las Heras, 2009), exposure to ultraviolet C (UVC: 200–280 nm), irradiation (Yin et al., 2013), soaking in hydrogen peroxide or washing in ethanol (Ginther, Issel‐Tarver, & King, 1992). The most frequently used technique was washing in sodium hypochlorite (bleach; Kemp & Smith, 2005)*.* These techniques were designed to eliminate exogenous DNA but their impact on endogenous DNA has not been established. The use of bleach was mentioned but limited. To extract teeth from the jaws under clean conditions, they should be brushed under tap water to remove dirt or debris and finally washed with DNA‐free water (Higgins & Austin, 2013).

The contamination of ancient samples resulting from contact with the environment is more difficult to detect than that resulting from laboratory works (Serre, Hofreiter, & Pääbo, 2004), in fact, the dental pulp was rarely contaminated by the environment due to its structure and protocol in teeth selection. With molecular analysis, it is necessary to use controls at the time of extraction and amplification of the DNA. These are either internal controls added at the time of extraction, to validate the extraction of the DNA at the time of amplification, or controls added at the time of amplification, to validate the amplification but not the extraction of the DNA (Handt, Höss, Krings, & Pääbo, 1994). The best way to eliminate doubts about laboratory contamination is the replication of results by another laboratory. It is actually the highest standard in active laboratories, especially in aDNA studies, to show true evidence (Willerslev & Cooper, 2005). For pathogen detection in ancient dental pulp, “suicide PCR” was considered as a principle to avoid any risk of contamination, it included: (a) primers which were used only once, (b) no positive control, (c) negative controls were added, (d) positive results were followed by sequencing. Although “suicide PCR” could show a low sensitivity, it had a high specificity and amplification process, ensuring almost positive predictive values (Raoult et al., 2000). It is important to follow correctly paleomicrobiology protocols for dental pulp to minimize risks of human DNA contamination during laboratory manipulation (Drancourt et al., 2017).

#### 3.1.7 Pulp recovery

There are several methods to collect ancient dental pulp. For instance, retrograde or ‘reverse root canal’ uses the root apex as penetration site (Cobb, 2002), while “orthograde entrance” followed the opposite direction by creating an orifice on the crown (Alakoç & Aka, 2009). Both methods almost preserve the morphological structure of the tooth. A third method has been applied in many research works: using an electric motor with rotating diamond disc to open the teeth longitudinally, afterward, the pulp and dentine powder is scraped into a sterile tube using a sterile instrument. The two parts of the tooth could be glued together after extirpation of the pulp in order to preserve dental morphology (Drancourt et al., 1998; La et al., 2004; Malou et al., 2012; Nguyen‐Hieu et al., 2010; Raoult et al., 2000, 2006; Tran, Forestier, et al., 2011; Tran, Signoli, et al., 2011).

The retrograde method that had the advantage of being minimally invasive to the tooth has been compared to grinding and spraying of the root or tooth. Although this technique created a hole for endodontic access, the amount of material retrieved was less important than with the grinding method, but the amount of amplifiable DNA per milligram of powder was significantly higher (Hughes‐Stamm, Warnke, & van Daal, 2016).

#### 3.1.8 Organic molecule extraction

**FIGURE 1:**
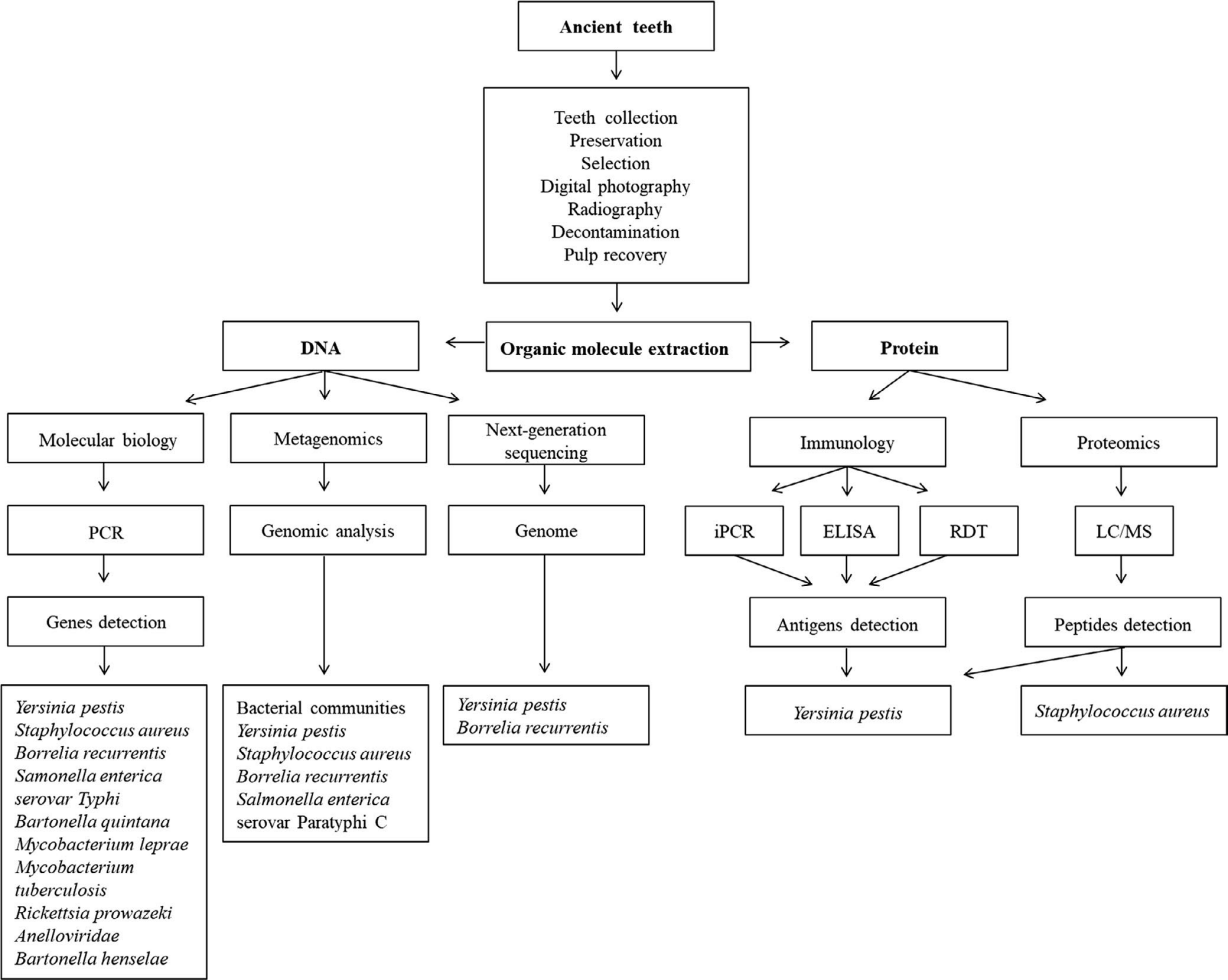
Methods for pathogens detection in ancient dental pulp

For the identification of pathogens, the DNA‐based detection approach is the most commonly used, while the emerging protein fragment‐based approach is increasingly used today and is very effective (Figure 1). It is efficient not only on ancient teeth but also on other ancient samples. Indeed, the phenol‐chloroform protocol has been widely used and has demonstrated optimal results in the recovery of nucleic acid with the highest quantity and quality (Barnett & Larson, 2012). For protein extraction, the modified protocol was applied for matrix‐assisted laser desorption ionization‐time of flight mass spectrometry (MALDI‐TOF MS), Enzyme‐Linked ImmunoSorbent Assay (ELISA), immuno‐Polymerase Chain Reaction PCR (iPCR), and Liquid Chromatography‐Mass Spectrometry (LC‐MS; Malou et al., 2012; Tran, Aboudharam, et al., 2011). Briefly, dental pulp powder was suspended into 200 µl of 0.5M EDTA (pH 8.0) solution with agitation at room temperature for 24 hr. After sonications and centrifugation at 17.900*g* for 40 min, the supernatant was stored at −20°C, the pellet was washed with distilled water and re‐suspended in 50 mM ammonium bicarbonate solution (pH 7.4) and incubated for 1 day, then centrifugation occurred at 17.900*g* for 40 min and the supernatant was collected. After dialyzing these fractions from EDTA and ammonium bicarbonate, proteins were concentrated and qualified by Bradford protocol (Bradford, 1976), and finally analyzed using the target techniques.

### 3.2 Methods for pathogens detection

#### 3.2.1 Molecular biology

##### Bacterial identification

Molecular detection is the most popular method to have a retrospective diagnosis by amplification of specific fragments of microbial genes in ancient human dental pulp. The most commonly used protocol is DNA extraction from dental pulp followed by PCR, sequencing and identification. The first demonstration of the presence of ancient bacterial DNA was the detection of *Y. pestis* using two molecular targets (Drancourt et al., 1998), most of the aDNA dated back to 4000 BC and found in human pulp belonged to *Bartonella quintana* (Drancourt, Tran‐Hung, Courtin, Lumley, & Raoult, 2005). From that time until now, many international research teams have used this method to discover multiple pathogens that were *B. quintana* (Drancourt et al., 2005; Nguyen‐Hieu et al., 2010; Raoult et al., 2006; Tran, Forestier, et al., 2011; Tran, Signoli, et al., 2011), *Salmonella enterica serovar Typhi* (Papagrigorakis, Yapijakis, Synodinos, & Baziotopoulou‐Valavani, 2006), *Mycobacterium leprae* (Matheson et al., 2009)*, Mycobacterium tuberculosis* (Matheson et al., 2009), *Y. pestis* (Bianucci et al., 2009; Drancourt et al., 1998, 2004, 2007; Haensch et al., 2010; Malou et al., 2012; Pusch, Rahalison, Blin, Nicholson, & Czarnetzki, 2004; Raoult et al., 2000; Schuenemann et al., 2011; Seifert et al., 2013, 2016; Tran, Forestier, et al., 2011; Tran, Signoli, et al., 2011; Wiechmann & Grupe, 2005), *Rickettsia prowazeki* (Nguyen‐Hieu et al., 2010; Raoult et al., 2006), *Staphylococcus aureus* (Drancourt et al., 2018) and *Borrelia recurrentis* (Guellil et al., 2018). Regarding animal teeth, *Bartonella henselae* was found in the dental pulp of cats dating from the 13th to the 16th centuries (La et al., 2004).

##### Viral identification

In contrast to bacteria for which several techniques are available, identification of viruses in ancient pulp was limited. Until now, only DNA of the family *Anelloviridae* was successfully confirmed from a soldier of Napoleon’s Army who died in Kaliningrad about 200 years ago (Bédarida, Dutour, Buzhilova, de Micco, & Biagini, 2011).

##### Bacterial genomes

Reconstruction of ancient bacterial genomes aims to elucidate the mechanisms of disease transmission in history and investigate the relationship to current emerging infections. Certainly, *Y. pestis* is the cause of plague disease, responsible for at least three high‐mortality pandemics in human history and many plague foci today, and about 40 historical burial places have been authenticated in Eurasia (Drancourt & Raoult, 2016). In 2011, the first draft genome was recovered from Black Death skeletons (Bos et al., 2011), after that, many ancient genomes from different periods were completed by next‐generation sequencing: nine genomes from the Black Death (Bos et al., 2011, 2016; Spyrou et al., 2016), three genomes from the Justinian Plague (Feldman et al., 2016; Wagner et al., 2014) and 15 genomes from about 1,500 to 3,000 years before the first plague recording (Andrades Valtueña et al., 2017; Rasmussen et al., 2015; Spyrou et al., 2018).

*Borrelia recurrentis* is the causative pathogen of louse‐borne relapsing fever (LBRF), this disease killed millions of people in European history and is now endemic in eastern Africa, its ancient genome was recovered in an individual from Oslo who died in the 15th century (Guellil et al., 2018).

#### 3.2.2 Metagenomics

Metagenomics is based on the association of bioinformatics and genomic technologies aimed at analyzing genes from communities of organisms. This method has more advantages than the molecular approach for aDNA studies because metagenomics was not influenced by length and sequence variants, even DNA fragments that were short or degraded could be successfully sequenced by High‐Throughput Sequencing (HTS) platforms (Warinner et al., 2017)*.* Metagenomics had access to this sample through analysis of 16S RNA amplicon sequencing data from dental pulp belonging to different chronological periods. The results indicated that ancient dental pulp could preserve the natural microbiome in the oral cavity and also in the dental pulp. In addition, this ancient specimen allowed DNA detection of oral pathogens that likely caused localized bacteremia (Rascovan et al., 2016). In 2018, a new metagenomic approach called MEGAN alignment tool (MALT) successfully analyzed data of DNA from ancient pulp and confirmed the presence of *Salmonella enterica* subsp. *enterica* serovar Paratyphi C, this pathogen was proposed as the causative agent of the epidemic at Teposcolula Yucundaa‐Mexico during the 1545–1550 period (Vågene et al., 2018). Metagenomics reaffirms and increases the diagnosis accuracy in many cases: *S. aureus* was found in a famous painter, Michelangelo Merisi ‘the Caravage’ (1571–1610) who died with signs of sepsis (Drancourt et al., 2018). *B. recurrentis* was confirmed in a 15th century skeleton (Guellil et al., 2018). *Y. pestis* was isolated from two 3800‐year‐old individuals: this is the oldest reported case of this bacterium in human pulp (Spyrou et al., 2018).

There are many factors linked to post‐mortem modifications that affected PCR amplification of ancient specimens. The presence of PCR inhibitors, including all substances that negatively impact on PCR results (such as decreased sensitivity or false‐negative) could be derived from the sample itself, during sample processing or DNA extraction (Schrader, Schielke, Ellerbroek, & Johne, 2012). The quality of aDNA was reduced, closely related to four damage types, each type has a different effect on DNA strands. The first and second types were oxidative damage and degradation, respectively, both caused strand break; the third was inter‐strand or inter‐molecular DNA crosslinks and the fourth was hydrolysis damage related to miscode. Moreover, modern DNA contamination is a great obstacle since PCR amplifies DNA fragments to obtain large quantities of copies (Rizzi et al., 2012).

Proteins that are non‐nucleotide biomolecules have proven to be very stable over time and can constitute a source of information in addition to nucleic acids in paleomicrobiology (Wadsworth & Buckley, 2014), the most ancient protein was isolated from a 68 million‐year‐old dinosaur (Schweitzer et al., 2007). For diagnosing the presence of pathogens in ancient tissues, pathologists used the immunological method that identified specific antigens of bacteria and definite methods that revealed the presence of bacterial proteins (Willcox, 2002).

#### 3.2.3 Immunology

##### Immunochromatographic assay

In 2003, a research group developed and successfully tested a new immunochromatographic assay (ICA), aimed at rapidly and exactly identifying *Y. pestis* in samples from people suspected of having plague. The technique used monoclonal antibodies to detect the F1 antigen of *Y. pestis*, as well as rapid diagnostic test (RDT) which was able to give the result after 10–15 min, positivity was defined when the threshold of the antigen concentration was higher than 0.5ng/ml and indicated by two pink lines (Chanteau et al., 2003). This method was applied to ancient human remains, including bone, and dental pulp to reveal that *Y. pestis* was the cause of the death of plague victims from different sites in Europe (Bianucci et al., 2008, 2009; Haensch et al., 2010)*.*

##### Immuno ‐ PCR (iPCR) and ELISA

Immunological methods with ELISA and immunohistochemistry were applied to identify F1 antigen of *Y. pestis* in ancient bone specimens (Cerutti, Marin, & Rabino Massa, 2007). Immuno‐PCR that was described in 1992 combined an immunological technique (ELISA) and a molecular technique (real‐time PCR), in which the marker was a specific DNA, it allowed increasing the sensitivity of detection of any specific antigen in samples (Sano, Smith, & Cantor, 1992). For the first time, both iPCR and ELISA were adapted for the detection of *Y. pestis* antigens in dental pulp, furthermore, combining three techniques (iPCR, ELISA, and PCR) gave the highest positive percentage in the diagnosis of 34 ancient teeth from five archeological sites (Malou et al., 2012).

##### Proteomics

Previously, bacterial identification based on host protein signatures was challenging for ancient specimens. For example*,* the presence of the agent of tuberculosis, *Mycobacterium tuberculosis*, in archaeological human remains was suggested by osteological examination and easily confirmed by biomolecular techniques. MALDI‐TOF MS, a new approach by proteomics, identified *M. tuberculosis* from human ancient bone samples through spectra of proteins (Boros‐Major et al., 2011; Hajdu et al., 2012) and mycolic acids (Mark, Patonai, Vaczy, Lorand, & Marcsik, 2010). Then, an article revealed that the presence of mycolic acids of *M. tuberculosis* in the previous research was not enough evidence to have confirmation (Minnikin, Lee, Pitts, Baird, & Besra, 2010). Furthermore, a study in 2016 showed the difficulties of protein identification from *M. tuberculosis* because of the lack of specific proteins, the peptide mass fingerprint used in these researches was not so different from human collagen (Hendy et al., 2016).

Recently, Liquid Chromatography‐Mass Spectrometry (LC/MS) was applied to determine four specific peptides of *Y. pestis* proteins from dental pulps in 3/16 individuals collected in archaeological plague‐positive places while in the negative control, no *Y. pestis* specific peptide was found. This result from LC/MS opens a new method in paleoproteomics that studies proteins to diagnose ancient blood‐borne pathogens in the dental pulp. Furthermore, 30 proteins of human origin were found in this research (Barbieri et al., 2017). The environment and time had a negative impact on proteins, meanwhile, a research in 2014 on current dental pulp discovered 342 proteins (Eckhardt, Jágr, Pataridis, & Mikšík, 2014). LC/MS confirmed once more the presence of *S. aureus* in the dental pulp of the painter Michelangelo Merisi (Drancourt et al., 2018).

## 4 DISCUSSIONS

**FIGURE 2:**
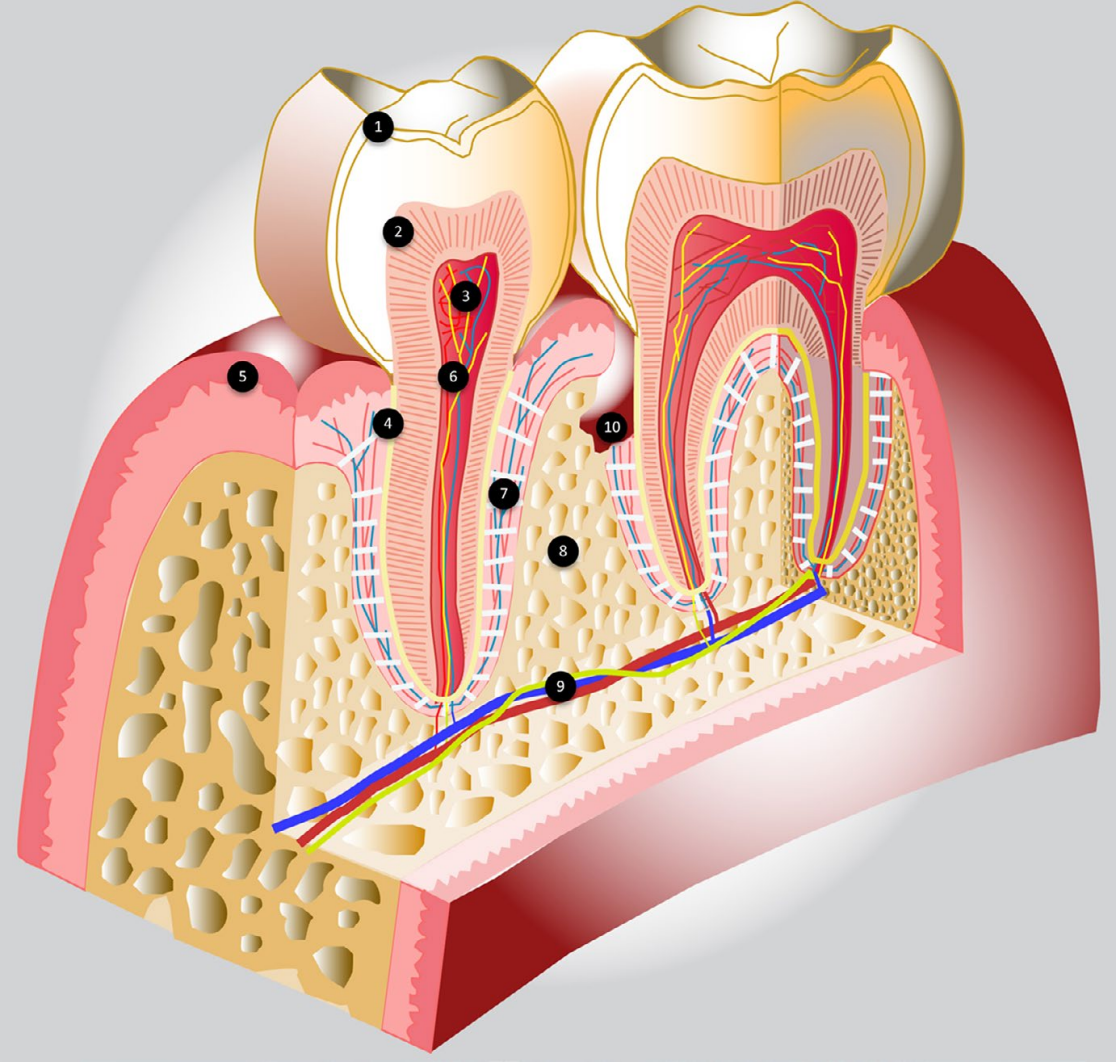
Schematic cross‐section of a premolar and a mandibular molar representing the different tissues. (1) Enamel, (2) Dentine, (3) Dental pulp, (4) Cementum, (5) Keratinized gum, (6) Blood vessel and nerves, terminale vascularization, (7) Periodontal ligament, (8) Alveolar bone, (9) Nerve and blood supply, (10) Periodontal pocket with dental calculus

To more understand the important role of the dental pulp in paleomicrobiology, we summarized its structural anatomy. The dental pulp is a specialized loose connective tissue present in the center of the tooth, derived from the neural crest, and situated within mineralized dentin that is covered by enamel on the crown and cement on the root. Histologically, typical pulp architecture, from the periphery to the center, consists of an odontoblastic zone, cell‐free zone or Weil zone, cell‐rich zone and the core of the pulp. The pulp contains numerous cell types such as odontoblasts, fibroblasts, immune cells, nerve cells, endothelial cells, and stem cells (Attoumani, Drancourt, & Ghigo, 2018; Huang, 2011). Blood vessels and nerve fibers enter the pulp via the root apex, creating a rich network with a high density. The blood circulation of the dental pulp is a terminal circulation with arteriovenous shunts that compensate for blood pressure (Figure 2). The smallest vessels form the terminal capillaries and are located within the odontoblastic layer. They influence the formation of dentin (Yoshida, Ohshima, & Kobayashi, 1988). The average capillary density in pulp is higher than in most other tissues with 1.402/mm^2^ (Vongsavan & Matthews, 1992) and the blood flow here is about 45 ml mm^−1^ 100 g^−1^ of tissue, nearly equal to that of the brain, lower than that of the heart and kidneys (Kim, 1985). The nerve fibers branch on the vessel walls and perivascular space. The neurovascular system maintains the normal function and self‐repair of teeth and has an important role in the regenerating process of the pulp tissue. Bacterial infection is considered the most common challenge to the pulp tissue (Kim et al., 2010). There are many ways for microbes to reach the pulp, listed as follows: exposed dentinal tubules, direct pulp exposure, periodontal pocket, faulty restoration, spread of an infected adjacent tooth, and blood‐borne microbes (Narayanan & Vaishnavi, 2010). Bacteria and their components are detected by pattern recognition receptors (PRRs) in which Toll‐like receptors (TLRs) play a central role for activating the innate immunity; these receptors are expressed on odontoblasts, fibroblasts, and stem cells. Binding these TLRs to bacterial antigens initiates an inflammatory reaction that activates cells and releases mediators (Fawzy El‐Sayed, Klingebiel, & Dörfer, 2016; Nakanishi et al., 2011; Yumoto et al., 2018). Adaptive immunity complements innate immunity. It creates immunological memory after initial response to specific antigens, includes lymphocytes, mast cells, cytokines, and chemokines. Both innate and adaptive immune mechanisms represent an important defense system that eliminates the effects of threatening factors (Attoumani et al., 2018; Hahn & Liewehr, 2007). After death, the pulp is degraded over time, the components and the products of this process are kept in the pulp cavity or on the surface of the dentin. Recuperating the pulp‐dentin complex or total tooth grind for investigating pathogens of ancient septicemia is essential for pulp analysis.

We suggested a general and detailed protocol with successive steps in using teeth for paleomicrobiology, from the collection at the beginning to organic molecule extraction at the final. Each step was established to ensure the authentic outcomes based on the analysis of DNA and protein. A total of seven methods was applied for pathogens’ identification, among them three for DNA (molecular biology, metagenomics,next‐generation sequencing) and four for protein (ICA, iPCR, ELISA and proteomics). Among the bacteria found in ancient dental pulp, *Y. pestis* has the largest number of publications, all bacterial identification methods were investigated for this pathogen, *S. aureus* and *B. recurrentis* were identified by three methods and others only by one (Figure 1). The availability of skeletal samples of plague has facilitated investigation, as evidenced by many methods created to increase diagnostic sensitivity, using proteins and DNA for material research. A general view of three methods was realized on ancient dental pulp, the percentage of detectable capacity is arranged here in increasing order: ELISA 8.8%, PCR 29.4%, iPCR 41.2%, and obviously, combining multiple methods increases the probability of bacterial detection in ancient pulp by 53% (Malou et al., 2012; Tables 2 and 3). The analysis of samples by autosomal short tandem repeats (STRs) shows that aDNA is better conserved in ancient dental pulp than in ancient bones (Ricaut, Keyser‐Tracqui, Crubézy, & Ludes, 2005). Although the quantity of DNA extracted was much lower in dental pulps than in ancient bones, the positivity rate confirmed using *Y. pestis pla* gene in qPCR from dental pulps was higher than for bone remains, with 5.7% in bones and 37% in teeth (Schuenemann et al., 2011). RDT was used to identify *Y. pestis* by targeting its specific protein, this technique gave a positivity rate of 35.7% (10/28 samples) while PCR was negative in 13 ancient bones samples (Haensch et al., 2010). RDT was applied to both ancient dental pulp and bones, which yielded different results. More specifically, bone and pulp samples were harvested from the same individuals, RDT detected *Y. pestis* with the rate of 50% in bones and 100% in pulp from four skeletons (Bianucci et al., 2009), another study showed the opposite result with 66.7% of positivity in bones and 19% of positivity in pulp from the same 21 skeletons (Bianucci et al., 2008).

**TABLE 2:** Summary of comparative results

*Yersinia pestis* diagnosis				
Percentage of positive teeth: iPCR: 41.2% PCR: 29.4% ELISA: 8.8% Combination of 3 techniques: 53% (Malou et al., 2012)	Ancient dental pulp	Quantity of DNA extraction (Schuenemann et al., 2011) < Amplification of DNA (Schuenemann et al., 2011) >	Ancient bone	RDT (+), PCR(−) (Haensch et al., 2010)
		RDT positive percentage (Bianucci et al., 2009) >		
		RDT positive percentage (Bianucci et al., 2008) <		

**TABLE 3:** Studies for pathogens detection in ancient dental pulp

Microorganism	Locations	Datation	Methods	Target	Teeth percentage	Reference
Pathogens detection in ancient dental pulp of human						
*Yersinia pestis*	Marseille ‐ France	1972	Standard PCR	Sequencing rpoB (133‐bp) gene and pla (300‐bp)	50% (4/8)	Drancourt et al. (1998)
	Lambesc ‐ France	1590			50% (2/4)	
	Vienna ‐ Austria	7−9th	Suicide nested PCR	Sequencing of glpD (191‐bp) gene	5.6% 2/36	Drancourt et al. (2007)
	Martigues ‐ France	1720–1721			2.8% (1/36)	
	Marseille ‐ France	1722			3.6% (2/56)	
	Montpellier ‐ France	14th	Suicide PCR	Sequencing pla (148‐bp) gene	86.9% (20/23)	Raoult et al. (2000)
	Venice – Italy	14th−16th	Real time PCR and suicide PCR	Sequencing pla (98‐bp) gene and glpD (191‐bp) gene	1.7% (3/173)	Tran, Signoli, et al. (2011)
	Aschheim ‐ Germany	6th	Suicide PCR	Sequencing pla (148‐bp) gene	33.3% (2/6)	Wiechmann and Grupe (2005)
	Bondy ‐ France	11th−15th	Real time PCR and suicide nested PCR	*pla* gene (98‐bp) and *glp*D gene (191‐bp)	28.6% (4/14)	Tran, Forestier, et al. (2011)
	Sens ‐ France Dreux ‐ France Montpellier ‐ France	541–767 1346–1800	Suicide PCR	Sequencing Multiple Spacer Typing	x/19	Drancourt et al. (2004)
	Manching ‐ Pichl ‐ Germany	1250–1500	Multiplex PCR	Sequencing Pla gene	4/20 individuals	Seifert et al. (2013)
	Brandenburg ‐ Germany	1640	Multiplex PCR	Sequencing Pla	3/3 individuals	
	London ‐ England	14th	Multiplex PCR	Sequencing Pla gene	36.9% (17/46)	Schuenemann et al. (2011)
	Bergen op Zoom ‐ The Netherlands	14th	Nested PCR	Sequencing pla (148‐bp) gene and caf1 (161‐bp) gene	16.3% (7/43)	Haensch et al. (2010)
	Hereford ‐ England	14th			16.7% (2/12)	
	Saint‐Laurent‐de‐la‐Cabrerisse ‐ France	14th			16.7% (1/6)	
	Lambesc ‐ France Draguignan ‐ France Berre l’Etang ‐ France Marseille ‐ France	16th−18th	RDT	*Y. pestis* F1 antigen	4.4% (4/91) 4.4% (4/91) 4.4% (4/91) 4.4% (4/91)	Bianucci et al. (2008)
	Hereford ‐ England	1335 ± 54			57.1% (4/7)	Haensch et al. (2010)
	Poitiers ‐ France	17th			X/7	Bianucci et al. (2009)
	La Chaize‐le‐Vicomte – France	17−18th			X/7	
	Bourges ‐ France Lariey ‐ France Sens ‐ France Bondy ‐ France Venice ‐ Italy	17th 5th−6th 11th−15th 14th−16th	Real‐time PCR		29.4% (10/34)	Malou et al. (2012)
			ELISA		8.8% (3/34)	
			iPCR		41.2% (14/34)	
	Le Délos ‐ France	18th	LC‐MS	Identifying four peptides at least *Y. pestis*	16.7% (3/18)	Barbieri et al. (2017)
	Brandenburg ‐ Germany	1618–1648	PCR sequencing	SNP analysis		Seifert et al. (2016)
	London‐England	1348–1350	NGS	1 genome		Bos et al. (2011)
	Marseille ‐ France	1722	NGS	5 genomes		Bos et al. (2016)
	Barcelona ‐ Spain	1300–1420	NGS	1 genome		Spyrou et al. (2016)
	Bolgar City ‐ Russia	1362–1400	NGS	1 genome		
	Ellwangen ‐ Germany	1485–1627	NGS	1 genome		
	Bavaria ‐ Germany	426 –571	NGS	1 genome		Feldman et al. (2016)
	Aschleim ‐ Germany	541–543	NGS	2 genomes		Wagner et al. (2014)
	Samara ‐ Russia	3800 BP	NGS	2 genomes		Spyrou et al. (2018)
	Bateni ‐ Russia	2909−2677 BC	NGS	2 genomes		Rasmussen et al. (2015)
	Sope ‐ Estonia	2575–2349 BC	NGS	1 genome		
	Bulavovo ‐ Russia	2280−2047 BC	NGS	1 genomes		
	Chociwel ‐ Poland	2135–1923 BC	NGS	1 genome		
	Kytmanovo ‐ Russia	1746–1626 BC	NGS	1 genome		
	Kapan ‐ Turkey	1048–855 BC	NGS	1 genome		
	Rasshevatskiy ‐ Russia	4828–4622 BP	NGS	1 genome		Andrades Valtueña et al. (2017)
	Beli Manastir, Popova zemlja ‐ Croatia	4833–4592 BP	NGS	1 genome		
	Gyvakarai ‐ Lithuania	4571–4422 BP	NGS	1 genome		
	Kunila ‐ Estonia	4524–4290 BP	NGS	1 genome		
	Augsburg ‐ Germany	4346–4098 BP	NGS	1 genome		
	Augsburg ‐ Germany	3957–3832 BP	NGS	1 genome		
*Bartonella quintana*	Peyraoutes ‐ France	4000 BC	Nested PCR	Sequencing of hbpE (283‐bp) gene and groEL (269‐bp) gene	16.7% (1/6)	Drancourt et al. (2005)
	Bondy ‐ France	11−15th	Real time PCR	ITS (102‐bp) gene	21.4% (3/14)	Tran, Forestier, et al. (2011)
	Venice ‐ Italia	15th−16th			2.9% (5/173)	Tran, Signoli, et al. (2011)
	Douai ‐ France	18th			2.5% (1/40)	Nguyen‐Hieu et al. (2010)
	Vilnius ‐ Lithuania	1812	Suicide nested PCR	Sequencing hbpE (282‐bp) gene and htrA (113‐bp) gene	9.7% (7/72)	Raoult et al. (2006)
*Mycobacterium leprae*	Jerusalem ‐ Israel	2025 ± 28 BP	Nested PCR	RLEP gene, 18kDa Antigen, ribosomal protein S12 IS6110	1/11 (9%)	Matheson et al. (2009)
*Mycobacterium tuberculosis*					3/11 (2.7%)	
*Salmonella typhi*	Athen ‐ Greece	430−426 BC	Suicide nested PCR	GosmC and clyA (322‐bp) gene and NarC (360‐bp) gene	3/3 (100%)	Papagrigorakis et al. (2006)
*Salmonella paratyphi C*	Teposcolula‐Yucundaa ‐ Mexico	1545–1550 CE	Metagenomic analysis		nd	Vågene et al. (2018)
*Borrelia recurrentis*	Oslo ‐ Norway	15th	Standard PCR Metagenomic analysis NGS	Unknown gene (111‐bp) 1 genome	2/18 (11.1%)	Guellil et al. (2018)
*Anelloviridae*	Kaliningrad ‐ Russia	1812	Standard PCR	Sequencing of non‐coding region of viral genome	1/21 (4.8%)	Bédarida et al. (2011)
*Rickettsia prowazekii*	Vilnius ‐ Lithuania	1812	Suicide nested PCR	Sequencing dnaA (141‐bp) gene and dnaE (77‐bp) gene	4/72 (5.6%)	Raoult et al. (2006)
	Douai – France	1710–1712	Suicide real‐time nested PCR + suicide PCR	Sequencing ipp (152‐bp), gap(130‐bp) genes and rpmEtRNAfMet intergenic spacer (115‐bp)	6/55 (10.9%)	Nguyen‐Hieu et al. (2010)
*Staphylococcus aureus*	Porto Ercole – Italy	1610	Non specific metagenomic + real time PCR + metaproteomic		nd	Drancourt et al. (2018)
Pathogen detection in ancient dental pulp of cat						
*Bartonella henselae*	Paris ‐ France	13th	Nested PCR	Sequencing groEL (269‐bp) gene and Pap31 (164‐bp)	1/4 cats	La et al. (2004)
	Montbéliard ‐ France	14th			1 cat	
	Compiègne ‐ France	16th			1/7 cats	

In total, 10 bacteria and one virus were identified by seven techniques in the human dental pulp during the last 20 years (Figure 1). However, there are still many other pathogens that have not been found in many investigations, responsible of epidemics in human history, such as *Bacillus anthracis* (anthrax; Nguyen‐Hieu et al., 2010; Papagrigorakis et al., 2006; Raoult et al., 2000, 2006; Tran, Forestier, et al., 2011; Tran, Signoli, et al., 2011), poxvirus (smallpox; Nguyen‐Hieu et al., 2010; Tran, Forestier, et al., 2011; Tran, Signoli, et al., 2011), cowpox virus (cowpox), and *Bartonella henselae* (cat‐scratch disease; Papagrigorakis et al., 2006). Therefore, it is useful to multiply multidisciplinary collaborations, not only to discover the microorganisms mentioned above but other microorganisms including viruses. In a previous study made from intact modern teeth, 100% healthy pulp (10/10), showed the presence of bacterial DNA. The predominant genera were Actinetobacter, Ralstonia, and Staphylococcus (Widmer et al., 2018). Until now, two virus that cause worldwide epidemics were found from modern pulpar tissues by molecular biology: human immunodeficiency virus (HIV; Glick, Trope, Bagasra, & Pliskin, 1991; Glick, Trope, & Pliskin, 1989) and hepatitis C virus (HCV), with a higher rate of identification of HCV in the dental pulp by molecular biology than in whole blood by immunoenzymatic reaction (Siravenha et al., 2016).

A lot of techniques have been applied to ancient materials but not ancient pulp. Immunohistochemistry uses monoclonal or polyclonal antibodies to determine antigens in tissue sections, a kind of antigen‐antibody reaction. By using this technique, *Treponema pallidum*, the causative agent of syphilis, was found on the skin of a female Italian mummy (1503–1568) in Naples, showing signs of tertiary syphilis (Fornaciari, Castagna, Tognetti, Tornaboni, & Bruno, 1989); *Rickettsia rickettsii*, which causes Rocky Mountain spotted fever (RMSF), was also identified in some ancient tissues from a patient who died in 1901 (Dumler, 1991). The immunologic techniques mentioned above belong to the field of paleoserology, including ICA, iPCR, and ELISA, which basically uses antibodies to detect *Y. pestis* antigens in the ancient pulp. Another technique consisting in the purification of immunoglobulin should be performed differently: immunoglobulins could be isolated from ancient pulp, after purification, then confronted to antigens of suspected pathogens. This method was practiced to confirm the presence of *T. pallidum* in a 200‐year‐old bone fragment (Kolman, Centurion‐Lara, Lukehart, Owsley, & Tuross, 1999).

Culture is the most common method in microbiology to isolate and identify microorganisms. However, that is a very difficult task to apply on ancient sources because of bacterial survival and suitable media of growth (Vishnivetskaya et al., 2006). Among bacteria found in ancient dental pulp, *Mycobacterium tuberculosis* can enter a dormant state during its life cycle and resists well to the environment and infected host. PCR testing for *M. tuberculosis* on samples of 300‐year‐old mummies yielded positive results but the culture method results were negative (Lemma et al., 2008).

In addition to confirming the aetiologies of blood‐borne infections, dental pulp also contributed to determine species, this is an important task that could involve genetic analysis or metagenomics. Recently, MALDI‐TOF MS allowed to distinguish species through creating protein files from various cell lines (Zhang, Scalf, Berggren, Westphall, & Smith, 2006). In 2011, this technique analyzed peptides from ancient dental pulp of different mammalian species, including humans, the specific spectrum of each species helped classify them into more accurate categories (Tran, Aboudharam, et al., 2011).

## 5 CONCLUSIONS

Since the dental pulp was recognized as a suitable sample for diagnosis of bacteremia in the past, many international research teams have used different techniques to detect pathogens with high sensitivity through nucleic acids and proteins. That contributed to clarify the cause of epidemics in history. Moreover, dental pulp helps to correctly classify ancient mammal species. All things considered, ancient pulp takes an important position in many different fields and will allow new discoveries in the future.
